# Case Report: Posterolateral Epidural Supra-C2-Root Approach (PESCA) for Biopsy of a Retro-Odontoid Lesions in Same Sitting After Occipitocervical Fixation and Decompression in a Case of Crowned Dens Syndrome With Brainstem Compression and Displacement

**DOI:** 10.3389/fsurg.2022.797495

**Published:** 2022-04-26

**Authors:** Patrick Haas, Till-Karsten Hauser, Kosmas Kandilaris, Marco Skardelly, Marcos Tatagiba, Sasan Darius Adib

**Affiliations:** ^1^Department of Neurosurgery, University of Tübingen, Tübingen, Germany; ^2^Department of Diagnostic and Interventional Neuroradiology, University of Tübingen, Tübingen, Germany; ^3^Department of Neuropathology, University of Tübingen, Tübingen, Germany

**Keywords:** crowned dens syndrome, PESCA, odontoid process, Saethre-Chotzen-Syndrome, pannus

## Abstract

**Background:**

‘Crowned dens syndrome' (CDS) is a special form of calcium pyrophosphate dihydrate deposition disease which is characterized radiologically by a halo-like or crown-like distribution in the periodontoid region and clinically by cervical pain. Herein, we will describe our experience of posterolateral epidural supra-C2-root approach (PESCA) for biopsy of retro-odontoid lesions in one surgical session after occipitocervical fixation and decompression in a patient with CDS and massive brainstem compression.

**Case Presentation:**

A 70-year-old woman presented to our department with a 4-week history of progressive walking impairment, neck pain, neck rigidity, fever, dizziness, slight palsy of the left hand, and multiple fall episodes. Magnetic resonance imaging (MRI) of the craniovertebral junction (CVJ) and cervical spine revealed a lesion of the odontoid process and the retro-odontoid region with mainly solid components, as well as small cystic components, and brainstem compression and displacement. In first step, fusion surgery of the CVJ C0–C4 was performed with occiptocervical decompression. After fusion and decompression the lower lateral part of the C1 arc and the lateral superior part of the left side of the C2 arc were removed. The entry point was located directly above the superior part of the C2 root. A biopsy of the lateral portions of the lesions was obtained by bioptic forceps under microscope guidance. Pathologic examination of the mass revealed deposition of birefringent crystals compatible with calcium pyrophosphate. In addition to the clinical symptoms (especially neck pain), the diagnosis of CDS was made. Non-steroidal inflammatory drugs (NSAIDs) and colchicine (and later magnesium) were started. At follow-up examination 6 months after surgery, an MRI scan of the cervical spine revealed regression of the pannus and the cyst with replacement of the brainstem, clinical improvement of walking, and increased strength of the left hand.

**Conclusions:**

This study demonstrates that PESCA can be used to obtain tissue for pathological analysis in one surgical sitting after fusion and decompression and that fusion, decompression, and PESCA (in the same session) together with subsequent conservative management could be a good alternative for the treatment of CDS.

## Introduction

Retro-odontoid pseudotumors are defined (1) as soft tissue proliferation at the atlantoaxial junction surrounding the region of the transverse ligament, and they might be associated with rheumatoid arthritis, microinstability, subluxation, as well as crystal deposition diseases (1).

Joyce et al. (2) pointed out that the term pannus is used in several medical contexts and that in rheumatology, pannus is defined as an “aggressive structure in the inflamed rheumatoid joint that invades cartilage and bone, thereby causing irreversible joint damage.” Pannus involves the atlanto-axial joint in rheumatoid arthritis and can cause instability and spinal cord injury due to compression of the cervicomedullary junction (2, 3), but it has also been used to describe retro-odontoid soft tissue masses in patients with juvenile idiopathic arthritis, spondyloarthritis, and calcium pyrophosphate dihydrate deposition (CPPD) disease (2).

Retro-odontoid pannus may develop in the spinal canal (4), may cause compression of the brainstem, may result in quadriplegia, or may even lead to sudden death in rare cases (5).

Crystal deposition diseases comprise a group of metabolic diseases, such as CPPD or hydroxyapatite crystal deposition (HAD), in which crystals are deposited in and around the joints and create inflammatory and destructive lesions (6).

While CPPD is the third most common inflammatory arthritis, characterized by acute (formerly known as pseudogout (7)) or chronic inflammation caused by deposits of CPP crystals in the articular cartilage and periarticular soft tissues, mostly in the knees and wrists (7), HAD is a systemic condition of unknown etiology (8).

In 1985, Bouvet et al. (9) first named a special form of CPPD (or HAD (10, 11)), which is characterized radiologically by a halo-like or crown-like distribution in the periodontoid region and clinically by cervical pain as 'crowned dens syndrome (CDS)' (6, 12–15). In most cases, CDS is managed conservatively, but in some rare cases with brainstem compression, myelopathy, and so on, surgery can be considered.

In a previous publication, we advocated for the posterolateral epidural supra-C2-root approach (PESCA) for biopsy of lesions of the odontoid process (OP) in one surgical session after occipitocervical fixation and decompression, which might be a good alternative to classical approaches (16).

Herein, we will describe our experience of PESCA for biopsy of retro-odontoid lesions in one surgical session after occipitocervical fixation and decompression in a patient with CDS and massive brainstem compression.

To best of our knowledge this is the first case of CDS which had been managed by this concept.

## Case Presentation

A 70-year-old woman presented to our department with a 4-week history of progressive walking impairment, neck pain, neck rigidity, fever, dizziness, slight palsy of the left hand, and multiple fall episodes.

**Figure 1:**
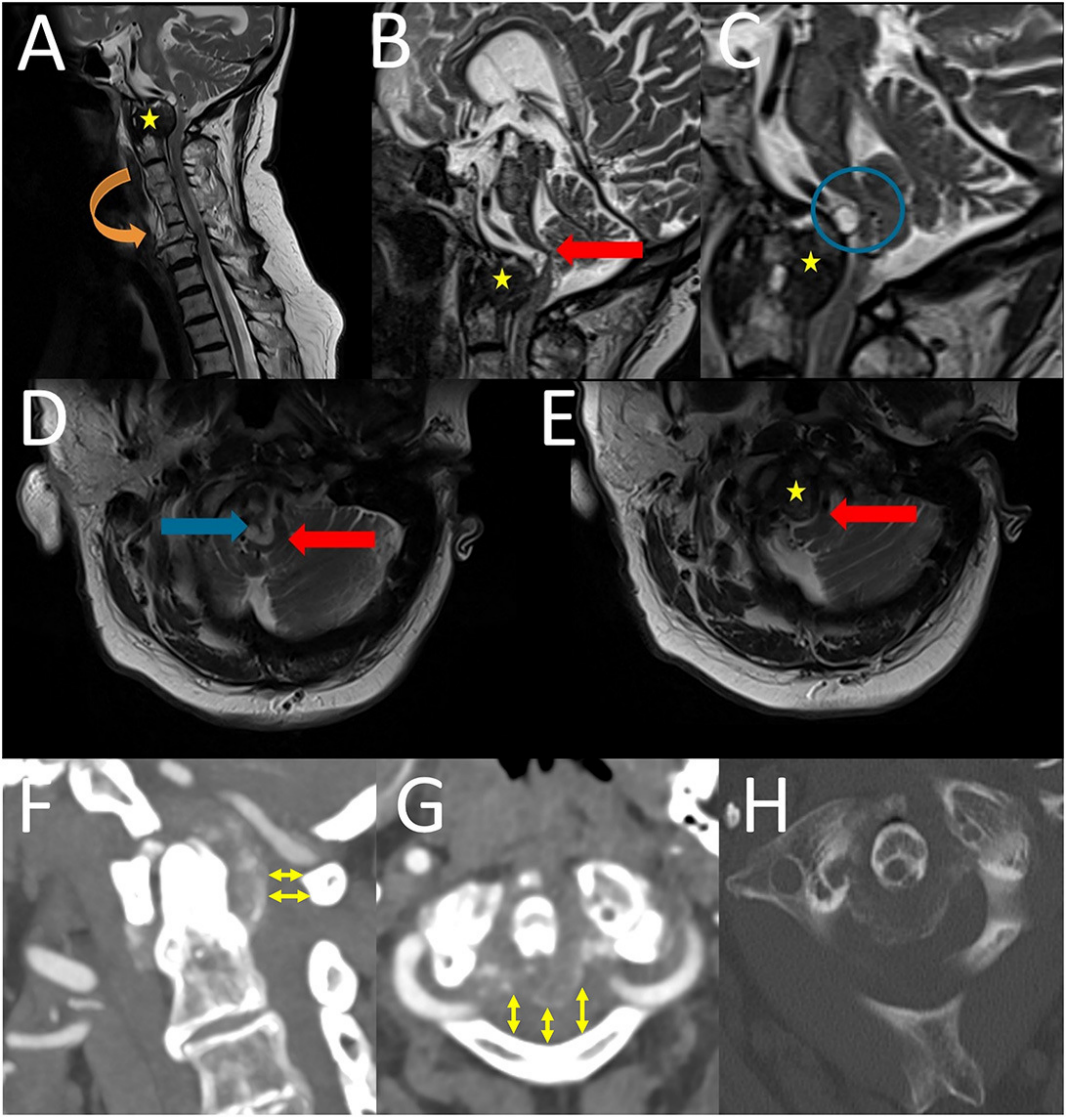
Preoperative sagittal **(A–C)** and axial **(D,E)** T2-weigthed magnetic resonance images revealed a lesion of the OP and the retro-odontoid region (yellow stars) with brainstem compression and displacement (red block arrows) and also cystic component (blue block arrows and blue circle). Furthermore, the cervical vertebral bodies C3 to C6 were fused due to Saethre-Chotzen syndrome (orange block arrow). Preoperative CT and CTA of cervical spine **(F–H)**: A sagittal **(F)** and an axial **(G)** CTA (soft tissue window) revealed a “normal” anatomy of the vertebral artery and furthermore a spinal stenosis in the level of C 1 (yelow arrows). An axial CT (bone window) showed a “horseshoe” or “crown-like” calcification, which is located posterior to the OP **(H)**.

Magnetic resonance imaging (MRI) of the craniovertebral junction (CVJ) and cervical spine revealed a lesion of the OP and the retro-odontoid region (Figures 1A,B) with mainly solid components, as well as small cystic components (Figure 1C), and brainstem compression and displacement (differential diagnoses include metastasis, rheumatoid arthritis, and spondylodiscitis) (Figures 1C,D). Computed tomography (CT) revealed spinal stenosis and odontoid erosion with signs of instability (Figures 1F–H).

The patient had a history of Saethre–Chotzen syndrome (SCS) (also known as acrocephalosyndactyly type III) and diabetes mellitus with diabetic nephropathy. MRI also revealed a fusion of the cervical vertebral bodies C3 to C6 due to Saethre-Chotzen syndrome (Figure 1A).

Owing to brainstem compression and displacement caused by pannus grade 4, cervical instability, and progressive walking impairment, we decided to perform surgery. Our goal was to stabilize the CVJ, decompress the foramen magnum and spinal canal at the C1 level, and perform biopsy of the periodontoid lesion for pathological analysis in a single surgical session.

### Intervention

Preoperative planning included a thin slice CT image of the cervical spine and CVJ for spinal neuronavigation, CT angiography (CTA) for analysis of the V3 segment of the vertebral artery, which revealed a “normal” anatomy, and a three-dimensional model print (1:1 scale model using the fused filament fabrication).

Cefuroxime was administered for perioperative surgical prophylaxis. The patient was placed in a prone position under general anesthesia, and radiography was performed after positioning to verify anatomical alignment. Intraoperative monitoring (IOM) included motor-evoked potentials and sensory-evoked potentials of the upper and lower extremities.

A midline incision was made and the inion, posterior wall of the posterior cranial fossa, C1 arc, and C2, C3, and C4 laminae were exposed.

Fusion surgery of the CVJ C0–C4 was performed with an OC plate (MOUNTAINEER, DePuy Synthes, Raynham, MA, USA) under spinal neuronavigation (Brainlab).

**Figure 2:**
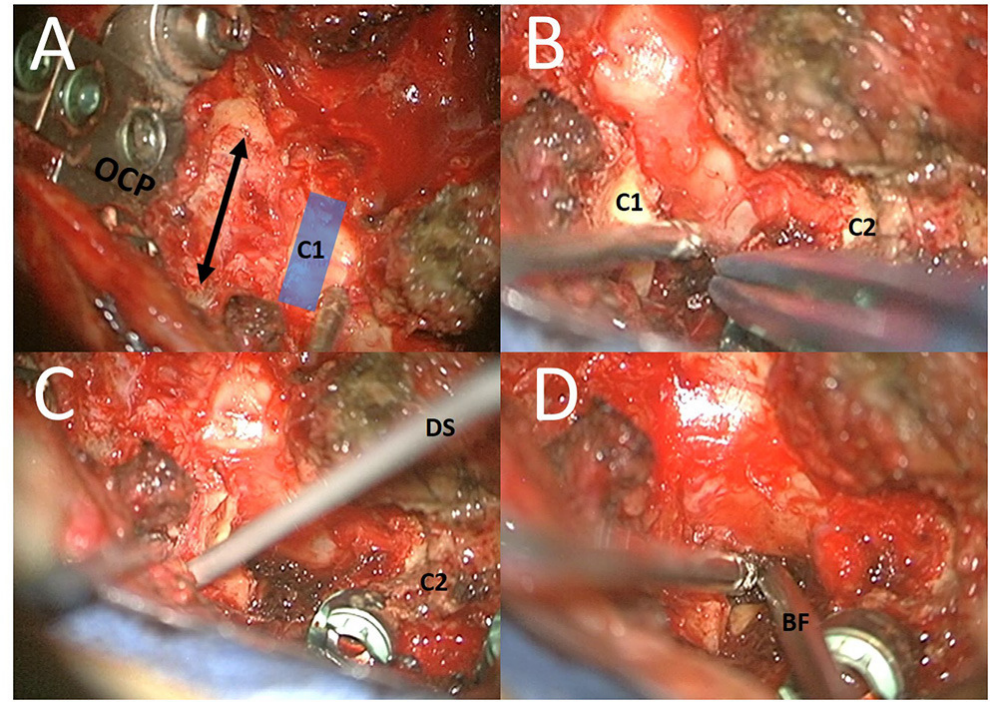
Different steps of surgery: **(A)** In first step, fusion surgery of the CVJ C0–C4 was performed with an OC plate (OCP) with occiptocervical decompression (by enlargement of the foramen magnum and laminectomy of the medial C1 arc); **(B,C)** After fusion and decompression the lower lateral part of the C1 arc (subperiostal, with remnant upper C1 arc) and the lateral superior part of the left side of the C2 arc. Doppler sonography (DS) was used to analyze the anatomy of vertebral artery. **(D)** The entry point was located directly above the superior part of the C2 root. The trajectory was located medial to the pedicle of C2, medial to the C1–C2 facet joint, and medial to the tubercle for the transverse ligament of the atlas. A biopsy of the lateral portions of the lesions was obtained by bioptic forceps (BF) under microscope guidance.

After fusion, the foramen magnum was enlarged under microscope (ZEISS KINEVO, Carl Zeiss, Germany) examination. Laminectomy of the medial C1 arc and the lower lateral part of the C1 arc (subperiostal, with remnant upper C1 arc), removal of the left superior part of the left side of the C2 arc, and flavectomy were performed (Figure 2). Doppler sonography was used to analyze the anatomy of vertebral artery (Figure 2).

Different landmarks such as the C2 root and remains of the C1 arc, C2 arc, and dural sac were identified. Then, PESCA, which we described in a previous publication, was performed (16).

The window between the remains of the C2 arc and the C2 root was used in our approach. The entry point was located directly above the superior part of the C2 root. The trajectory was located medial to the pedicle of C2, medial to the C1–C2 facet joint, and medial to the tubercle for the transverse ligament of the atlas.

A bioptic instrument was inserted under microscope guidance (Figure 2). Owing to dorsal decompression, the danger of compression was limited as much as possible. IOM remained stable during surgery. A biopsy of the lateral portions of the lesions was obtained.

### Postoperative Course

**Figure 3:**
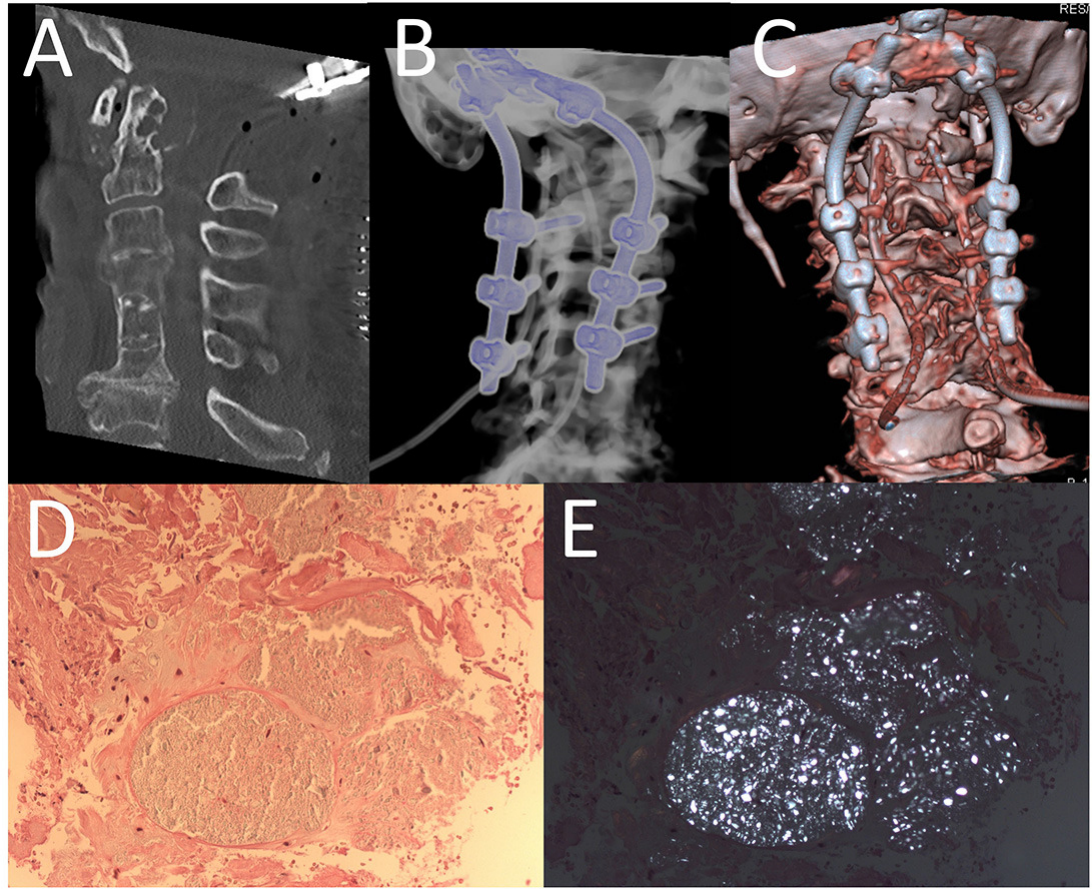
Post-operative CT-scan of cervical spine **(A)** and different three-dimensional reconstruction **(B,C)** revealed a sufficient decompression and furthermore regular placement of screws. Histopathologic findings of bioptic probe showing fibrous connective tissue (HE x 200) **(D)** with multifocal deposits of birefringent crystals (under polarized light x 200) **(E)**.

A postoperative CT scan showed proper positioning of the screws and sufficient decompression of the spinal cord at the level of the CVJ (Figures 3A–C). The patient recovered from surgery without any new deficits.

Pathologic examination of the mass revealed fibrous connective tissue with deposition of birefringent crystals compatible with calcium pyrophosphate (CPP) due to CPPD disease (Figures 3D,E). In addition to the clinical symptoms (especially neck pain), the diagnosis of CDS was made. Non-steroidal inflammatory drugs (NSAIDs) and colchicine were started.

### Follow-Up

At follow-up examination 3 months after surgery, the patient did not manifest any neurological symptoms, and the CT scan of the CVJ did not reveal large regression of the pannus. Therefore, NSAIDs (diclofenac), steroids (prednisolone), and magnesium were administered.

**Figure 4:**
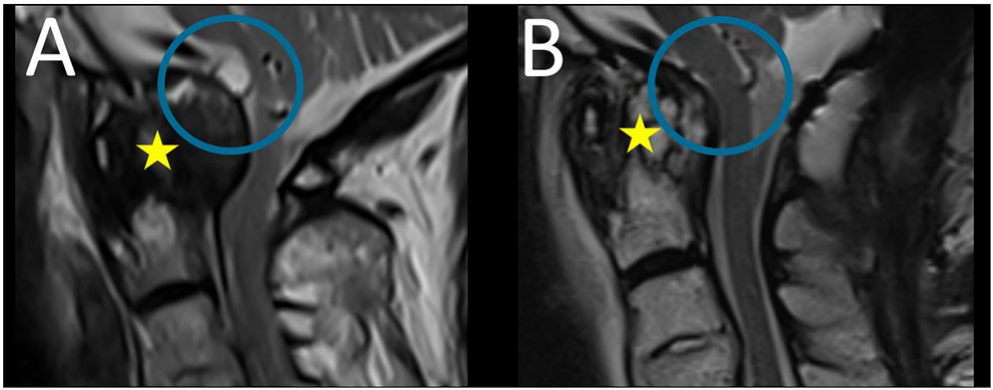
Preoperative **(A)** and follow-up **(B)** sagittal T2-weigthed magnetic resonance images: follow-up MRI scan revealed regression of the pannus (yellow star) and the cyst with replacement of the brainstem (blue circle).

At follow-up examination 6 months after surgery, an MRI scan of the cervical spine revealed regression of the pannus and the cyst with replacement of the brainstem (Figure 4), clinical improvement of walking, and increased strength of the left hand. Since then, the patient did not experience neck pain and dizziness.

## Discussion

### Crowned Dens Syndrome

Oka et al. (17) summarized in their review 72 published cases of CDS (including their own three cases) and found that the mean patient age was 71.4 (26–93) years, 47.2% of the patients were male, 52.8% were female, and 54.1% had peripheral arthritis. Furthermore, they concluded that the classical triad of CDS is neck pain (100%), neck rigidity (98%), and fever (80.4%). Besides these symptoms, 19.1% of the patients had shoulder pain and 8.3% experienced occipital or temporal pain (17). Myelopathy was detected in 5.5% of the cases (17).

The precise diagnosis of CDS might be challenging (17–19), as the symptoms are similar to those of other diseases, such as spondylodiscitis, meningitis, cervicobrachial pain, polymyalgia rheumatism, occipitotemporal headache, giant cell arteritis, calcific tendinitis of the longus colli muscle, and retropharyngeal abscess.

### Radiological Diagnosis

Different authors concluded that CT of the CVJ is the gold standard for the diagnosis of CDS (13, 17). The goal is to detect the horseshoe or crown-like calcification (13), which is located posterior to the OP in approximately 90% of cases (19), but also might be located in different structures around the OP such as the transverse ligament, alar, and cruciate ligaments, articular capsule, and synovial membrane (13). Another typical CT finding is the combination of subchondral cysts and erosion in the OP (19), similar to our case.

Jain et al. (19) concluded that the retro-odontoid pseudotumor in case of crystal deposition appears hypointense to marrow signal on both T1- and T2-weighted images, compressing the odontoid. There may be further degenerative changes such as sclerosis, osteophytosis, and subluxation (19).

Grob et al. (5) described four grades of pannus: grade 1, little/no pannus; grade 2, moderate pannus; grade 3, massive pannus, without spinal cord compression; grade 4, massive pannus with spinal cord or brain stem compression. In our case, the patient had a grade 4 pannus.

Different types of pannus have been classified in the literature such as hypervascular, hypovascular, and fibrous pannus (1).

### Pathophysiology of Calcium Pyrophosphate Dihydrate Deposition Disease

Many authors (7, 20) presented that the pathomechanism of CPPD crystal formation in the articular fibrocartilaginous structures has not been completely understood yet. Several pathophysiological theories exist about CPPD. CPPD may result from an imbalance between the production of pyrophoshate and the level of pyrophosphatases in the diseased cartilage (21). Pyrophosphate deposits in the synovium may combine with calcium to form CPP crystals (7). The formation of CPP crystals in the pericellular matrix of the cartilage is the first step in the disease process (7), but chondrocytes appear to play an important role.

Chondrocytes generate “pericellular exosome-sized vesicles,” also termed as “articular cartilage vesicles,” which are one of the important sites of crystal formation in cartilage; furthermore, they produce extracellular inorganic phosphates, which are essential to the formation of CPP crystals (7).

Zünkeler et al. (20) hypothesized that fibroblast in the cervical spine ligament transforms into chondrocyte and that the transformation causes calcification. Furthermore, they postulated that mechanical trauma may be the initial event that affect crystal formation.

Once located in the joint, CPP crystals may contribute to further mechanical damage (by altering the mechanical properties of the cartilage (7)) of the adjacent joint tissue and initiate an inflammatory process by activating components of the NLRP3 inflammasome and by creating neutrophil extracellular traps (7), as suggested by experimental studies in which CPP crystals were injected into the synovial space (20, 22).

Moreover, a number of comorbidities correlate with CPPD (21). Different studies demonstrated that hyperparathyroidism presented the highest positive association with CPPD, followed by gout, osteoarthritis, rheumatoid arthritis, and hemochromatosis (21). Beside these comorbidities, hypomagnesemia, osteoporosis, chronic kidney disease, calcium supplementation (21), and Wilson's disease (20, 23) appear to be related.

### Conservative Treatment Options of CDS

Most authors have recommended treatment with NSAIDs and/or steroids (especially prednisolone) (13, 24, 25). Oka et al. (17) summarized in their review that 85% of the patients with CDS were treated with NSAID alone or NSAID with another drug. In most of the cases, the clinical symptoms improve within 4–7 days (11, 13, 26).

Lee et al. (13) pointed out that after the initial improvement within 1 week, there is a slow but persistent improvement in 3–5 weeks.

Oka et al. (17) reported that 67.5% of patients with CDS were treated with NSAID alone, 15% with steroids alone, 7.5% with NSAIDs and steroids, 5% with NSAID and tizanideine, 2.5% with NSAID, colchicine, and steroid, and 2.5% with NSAID and carbamazine.

Different authors (7, 17) also presented the use of magnesium, iron chelators, probenecid, and phosphocitrate for the treatment of associated metabolic conditions in patients with CPPD (especially to inhibit crystal formation) and colchicine, methotrexate, and hydroxychloroquine to prevent the inflammasome activation. Treatment with interleukin-1 (IL-1) inhibitor is possible (e.g., anakinra, canakinumab, IL-1 trap).

Jain et al. (19) advocated the use of NSAIDs and colchicine and mentioned that patients show dramatic improved during this treatment.

As shown in the present case and in some cases, treatment of pannus by conservative methods may take months. During this time, the patient is already at a high risk of further impairment, especially in the presence of brainstem compression.

### Surgical Treatment of CDS

Even if conservative therapy is indicated in most cases of CDS, surgery is also indicated in a few cases (27). Fiani et al. (4) concluded that occipital–cervical fusion “is indicated in cases where the panni impinge on the medulla and the upper cervical cord” and that the “goal in occipital surgical fusion is to prevent further progression of the pseudotumor and improve neurological outcomes.” Furthermore, they concluded that “neurological improvements are often noted in patients as soon as 1 week after surgery and complete resolution of the pseudotumor can be visualized on imaging within 1 year of surgical repair.”

Baysal et al. (28) reported that among 17 patients of CDS who progressively presented neurological symptoms, one patient was treated by decompression surgery. Zünkeler et al. (20) performed surgery in six of seven patients with periodontoid CPPD disease, and most of them even underwent two surgical sessions: first with the transoral–transpharyngeal approach and second with the posterior fusion of C0–C2.

According to most authors and to our opinion, surgery is necessary in case of massive brainstem compression, myelopathy, dramatic progression of neurological symptoms, unclear diagnosis (e.g., in case of DD metastases) and instability. In our case, the patient had a massive brainstem compression and displacement and progressive walking impairment. Therefore, surgery was performed.

PESCA might be a good alternative and is easier to perform in periodontoid lesions than in odontoid lesions because the trajectory is not as deep. Even if the window of PESCA is small, the surgical path is narrow, and the working angle is oriented up, performing surgery in one session is a huge advantage for the patient (16). To best of our knowledge this is the first case of CDS which had been managed by this concept.

### Craniocervical Junction Abnormalities in Saethre-Chotzen Syndrome

In this report, the patient had SCS, which is a craniosynostosis syndrome that arises in 1 per 100,000 live births (29). It presents as low hairline, ptosis, external ear abnormalities, tear duct stenosis, hand anomalies, and short statute. Clinical diagnosis in these patients is usually genetically confirmed by a deletion of mutation in the *TWIST1* gene (29).

Cervical spinal changes have been described in SCS. Anderson et al. (30) and Trusen et al. (31) reported that fusion of vertebral bodies and/or posterior elements may occur in the cervical spine.

### Lateral and Posterior Approaches to OP

Riley et al. (32) concluded that there are three approaches to the OP: 1. Anterior, 2. Lateral and 3. Posterior.

Beside anterior approaches (such as transoral, endoscopic endonasal, anterior high retrophayryngeal and transcervical approaches) (32, 33) several authors have advocated for the lateral and for the posterior approaches:

A number of authors (34–36) described the (far lateral) transcondylar approach, the trans-atlas extradural approach, the extreme lateral-transatlas approach (37), and the extreme lateral trans-odontoid (ELTO)^35^ used in the removal of OP, retro-odontoid lesions (such as synovial cysts) or extending lesions in and around the OP (such as chordomas).

One risk of transcondylar and trans-atlas approaches is instability (37). Another risk of transcondylar approach is injury of hypoglossal nerve due to proximity in its location (38, 39). On the-other-hand trans-atlas approach includes the risk of injury of the VA (37).

Oya et al. (40) described an approach with skin incision on the posterior margin of the sternocleidomastoid muscle. Then, they cut a reflection of the SCM to be inserted in the posterior space of the SCM muscle, transverse process of C1, C2, and C3, in order for odontoidectomy to be carried out. Naito et al. (41) published the high cervical lateral approach through retroauricular curved skin incision for removal of retro-odontoid pseudotumors.

Srivastava et al. (42) described a simultaneous odontoid excision with bilateral posterior C1-2 distraction and stabilization utilizing bilateral posterolateral corridors and a single posterior midline incision. Grundy et Gill described an approach to OP through a midline incision from the external occipital protuberance to the spinous process of C6, and a transverse occipital incision (T-Incision). The posterior arch of C1 was removed as well as the pedicle of C1 and posterior boundary of the vertebral canal.

The posteriolateral transpedicular approach to C2 has a narrow trajectory (because of the diameter and angle of the pedicles); therefore, the reachable targets are limited and in most cases, the upper part of the OP is not reachable. This approach has been used mainly for biopsy.

Riley et al. (32) advocated for the METRx posterolateral approach, which uses a paravertebral incision and they entered a METRx dilatator for a minimal invasive surgical approach to OP. Eissa and Eldin (43) analyzed an approach in which they performed a midline skin incision on cadavers and extended it laterally (as inverted L) to help the lateral dissection and exposure of the vertebral artery. A C2 neurectomy was perfomed with exposure of the C2 *pars interarticularis* and the inferior articular atlas was used as a guide to expose the atlanto-occipital joint ways. Mobilization of VA could be necessary to enlarge the surgical window (44).

The most posterior approach is the transdural approach (45, 46) which has a high risk of cerebrospinal fluid leakage and infection. Furthermore, in the case of a tumor or infection, the dura mater (a natural barrier) is opened and may lead to intradural insertion of the pathology.

Main advantage of posteriolateral approaches is that occipitocervical fixation and decompression can be performed in same sitting (16).

In our case we used PESCA (16) which uses a midline incision in combination with previous decompression, thereby enlarging the foramen of magnum and medial C1 removal. The lateral corridor between the lateral part of C1 arch and the lateral part of C2 arch is enlarged by drilling of the inferior lateral part of the C1 arch and the lateral superior part of C2 arch. The condyles and the atlas were not removed.

## Conclusion

In summary, CDS is a rare disease that usually can be treated conservatively. In cases of brainstem compression, brainstem displacement, or neurological impairment, surgery should be discussed to prevent further worsening of neurological symptoms or even death.

To best of our knowledge, SCS and CDS in the same patient have not been described yet. A correlation of CDS and SCS has been not described in literature. This study demonstrates that PESCA can be used to obtain tissue for pathological analysis in one surgical sitting after fusion and decompression and that fusion, decompression, and PESCA (in the same session) together with subsequent conservative management could be a good alternative for the treatment of CDS.

## Data Availability Statement

The original contributions presented in the study are included in the article/supplementary materials, further inquiries can be directed to the corresponding author.

## Ethics Statement

The studies involving human participants were reviewed and approved by Ethics Committee of the University Hospital Tübingen, Germany; reference number 478/2020BO. The patients/participants provided their written informed consent to participate in this study.

## Author Contributions

PH: performed analyses, performed 3D-print, and critical revision. T-KH, KK, MS, and MT: performed analyses and critical revision. SA: idea and development of PESCA, performed surgery, performed analyses, wrote the article, and critical revision. All authors listed have made substantial, direct, and intellectual contribution to the work and approved it for publications.

## Funding

We acknowledge support by Deutsche Forschungsgemeinschaft and Open Access Publishing Fund of the University of Tübingen.

## Conflict of Interest

The authors declare that the research was conducted in the absence of any commercial or financial relationships that could be construed as a potential conflict of interest.

## Publisher's Note

All claims expressed in this article are solely those of the authors and do not necessarily represent those of their affiliated organizations, or those of the publisher, the editors and the reviewers. Any product that may be evaluated in this article, or claim that may be made by its manufacturer, is not guaranteed or endorsed by the publisher.
